# Free will and trolley dilemmas: evidence for moral inertia in a Venezuelan sample

**DOI:** 10.3389/fpsyg.2026.1748028

**Published:** 2026-01-30

**Authors:** Gabriel Andrade, Laura Gamboa, Maria Campo-Redondo

**Affiliations:** 1 Ajman University, Ajman, United Arab Emirates; 2 Universidad del Zulia, Maracaibo, Venezuela; 3 United Arab Emirates University, Al-Ain, United Arab Emirates

**Keywords:** agency beliefs, determinism, free will, moral judgment, trolley problem

## Abstract

**Background:**

Beliefs about free will are central to philosophical and scientific conceptions of agency, and experimental work suggests that weakening such beliefs can reduce honesty, self-control, and helping. Yet little is known about how disbelief in free will influences moral reasoning in classic dilemmas contrasting utilitarian and non-utilitarian responses.

**Methods:**

Three randomized studies were conducted with Venezuelan university students (*N* = 88 per study). Participants read either an adapted deterministic passage adapted from Crick or a neutral neuroscience text, then responded yes/no to the Spur, Footbridge, or Singer “Drowning Child” dilemmas. Fisher’s exact tests, with follow-up logistic regressions, assessed effects of condition on moral choices.

**Results:**

Responses showed the expected baseline patterns across dilemmas. The determinism manipulation reduced willingness to intervene in the Spur dilemma (*p* = 0.0385, fewer participants pulled the switch) and reduced willingness to help in the Singer scenario (*p* = 0.0261), but had no detectable effect on Footbridge judgments (*p* = 0.783).

**Conclusion:**

Inducing disbelief in free will appears to reduce proactive moral intervention rather than increasing willingness to endorse direct personal harm.

## Introduction

Belief in free will has held a central place in Western thought, from early theological debates to modern discussions in philosophy and neuroscience. The question of whether human decisions are truly free or determined by prior causes remains unsettled. Thinkers like Hume, Kant, and Strawson offered sharply differing views on whether agency is real, constrained but compatible with causality, or an adaptive illusion (McFee, 2014). Recently, psychologists have moved this debate from the realm of metaphysics to empirical inquiry, exploring how beliefs about free will shape everyday behavior and moral judgment.

While philosophers ask whether free will exists, psychologists ask what happens when people believe—or stop believing—in it. Studies by Vohs and Schooler (2008), Schooler et al. (2014), and Baumeister and Brewer (2012) show that weakening belief in free will can affect honesty, self-control, and prosocial motivation. Reading deterministic texts has been found to increase cheating or reduce helping, suggesting that agency beliefs regulate moral conduct. People who see themselves as autonomous feel more accountable for their actions; those who adopt a deterministic view may experience reduced responsibility. However, how these beliefs alter moral reasoning remains uncertain—whether mainly through cognitive changes in weighing consequences or through emotional shifts in empathy and guilt.

While previous studies have assessed the effects of diminished belief in free will on cheating, aggression, and helping, little is known about how this manipulation influences reasoning in structured moral dilemmas. Moral psychology has developed rich paradigms for studying such reasoning, notably the trolley problems, which directly pit utilitarian cost–benefit calculations against non-utilitarian constraints forbidding personal harm (Andrade, 2019). Understanding whether belief in free will shapes how individuals resolve these trade-offs may illuminate the moral functions of agency beliefs beyond simple behavioral measures.

The trolley problem, introduced by Philippa Foot and later refined by Judith Jarvis Thomson, exemplifies moral conflict between outcomes and duties (Edmonds, 2015). In the Spur version, a bystander can pull a lever to divert a trolley, sacrificing one to save five—a largely impersonal, utilitarian trade. In the Footbridge version, the only way to save the five is to push a large man onto the tracks, a more emotionally charged and personal decision. People typically pull the lever but refuse to push, showing that non-utilitarian aversion to direct harm often overrides utilitarian reasoning (Nyholm, 2023).

These dilemmas provide a useful framework for testing how disbelief in free will shapes moral thought. Weakening belief in free will might encourage a mechanistic, result-focused perspective—or, conversely, reduce willingness to act when harm is involved, since denying agency could diminish personal responsibility. Such effects extend beyond the utilitarian/non-utilitarian divide, potentially reshaping perceptions of moral authorship itself: whether one feels like an active agent or a passive observer of determined events.

From this perspective, the present research examined whether inducing disbelief in free will changes responses to moral dilemmas. The study’s research question is whether reduced belief in free will leads people to alter utilitarian judgments—or whether the change reflects a broader shift in moral engagement and sense of responsibility.

## Methods

Three independent studies were conducted with distinct samples of Venezuelan university students to minimize framing or contextual effects across moral dilemmas. All protocols received institutional ethics approval and followed the Declaration of Helsinki. Participants gave written informed consent and reported age and gender for potential control in analyses.

Power analysis using G*Power 3.1 (w = 0.3, *α* = 0.05, power = 0.80) indicated a target of 88 participants per study. Recruitment occurred via campus notices in a University in Venezuela. In each study, participants were randomly assigned in equal numbers to experimental or control groups, ensuring comparable baseline characteristics and internal validity.

Participants assigned to the experimental (determinism) condition read an adapted Spanish-language excerpt from Crick (1994) *The Astonishing Hypothesis* (in a format easier to understand) in which the notion of free will is explicitly questioned and human behavior is described as determined by neural mechanisms. For this task they were given 25 min. This is done following the guidelines of Vohs and Schooler (2008) in their study of the effects of belief in free will. Immediately after reading, to reinforce the manipulation, participants summarized the main points of the passage in their own words, by writing a summary; for this task, they were given 20 min. This well-established procedure was employed consistently across all three studies as a determinism-themed prime that is intended, on theoretical grounds and prior evidence, to reduce belief in free will and personal agency, although the present studies did not include a direct manipulation check of that change.

Control participants received a text of similar length about general brain structure and cognitive functioning, devoid of any references to free will, agency, or determinism. They were likewise asked to summarize the main points, equating the cognitive demands of the manipulation across conditions and ensuring that only the content relating to free will belief differed between groups. Timing was the same as in the experimental condition.

The critical dependent variable was participants’ responses to moral dilemmas presented 5 min after the reading and summarization tasks. Participants were told there would be a simple moral task. In Study 1, the classic “Spur” (trolley) dilemma was used: participants indicated whether they would pull a lever to divert an out-of-control trolley, sacrificing one person to save five. In Study 2, the “Footbridge” version was employed, in which the decision was whether to push a large individual from a bridge to stop a trolley and save five. Study 3 adapted Peter Singer’s famous “Drowning child” thought experiment, presenting participants with the choice of jumping into a pond to save a drowning child at the cost of ruining their expensive shoes (Singer, 2016). All outcomes were binary (yes/no).

For each study, the primary analysis of the effect of experimental manipulation on moral judgment was conducted using Fisher’s exact test on 2 × 2 contingency tables, comparing experimental condition with participants’ dichotomous responses to the moral dilemma. Fisher’s exact test was chosen due to the moderate sample sizes and the possibility of small cell counts. In studies where this test produced significant results, secondary logistic regression analyses were conducted including age and gender as covariates, to evaluate the robustness of the effect while controlling for potential demographic confounders.

## Results

Across the three studies, participants’ responses followed the expected patterns for each dilemma. In Study 1 (Spur), 74 of 88 students (84.1%) chose the utilitarian option of pulling the lever, while 14 (15.9%) chose the non-utilitarian alternative. In Study 2 (Footbridge), the pattern reversed: 72 of 88 (81.8%) rejected pushing the person, and 16 (18.2%) endorsed the utilitarian choice. In Study 3 (Drowning Child), 82 of 88 (93.2%) agreed to save the child, while 6 (6.8%) refused. These data confirm the expected response profiles—utilitarian for the impersonal dilemma, non-utilitarian for the personal one, and predominantly prosocial for the Singer-style case.

Demographic characteristics were comparable across studies. Gender was balanced in Studies 1 and 2 (roughly 50% male and female), and Study 3 included 49 women (55.7%) and 39 men (44.3%). Mean ages were 22.7 (SD = 3.00), 21.1 (SD = 1.84), and 22.4 (SD = 2.05) years, respectively, with ranges from 18 to the mid-20s. These similarities minimize the likelihood that demographic factors explain the observed effects.

In Study 1, the free-will manipulation affected responses to the Spur dilemma. Among those exposed to the determinism passage, 33 chose the utilitarian option and 11 the non-utilitarian one, compared with 41 utilitarian and 3 non-utilitarian responses in the control group. Fisher’s exact test showed a significant difference (*p* = 0.0385; OR = 0.22, effect size phi = 0.25). Logistic regression with condition, age, and gender confirmed that condition remained significant (*p* = 0.034), while age and gender were not. The effect was modest (McFadden’s pseudo-*R*^2^ = 0.0772) but indicated that deterministic primes increased the likelihood of omission in the impersonal scenario (participants were less likely to pull the lever to intervene).

Study 2 showed no such effect on the Footbridge dilemma. In the experimental group, 37 participants gave non-utilitarian and 7 utilitarian responses, compared to 35 and 9 in the control group. Fisher’s exact test was non-significant (*p* = 0.783; OR = 0.74, effect size phi = 0.2), so no adjusted model was estimated. The null result suggests that challenging belief in free will does not alter reluctance to directly cause harm.

In Study 3, 38 participants in the experimental group and 44 in the control group chose to save the child, while 6 in the experimental group and none in the control group refused. Fisher’s test showed a significant association (*p* = 0.0261; OR = 0.07, effect size phi = 0.26). Yet logistic regression including age and gender found no significant predictors. The overall model fit (McFadden’s pseudo-*R*^2^ = 0.205) was limited due to sparse “no-help” responses, especially the zero frequency in the control group, producing unstable coefficients.

**Figure 1:**
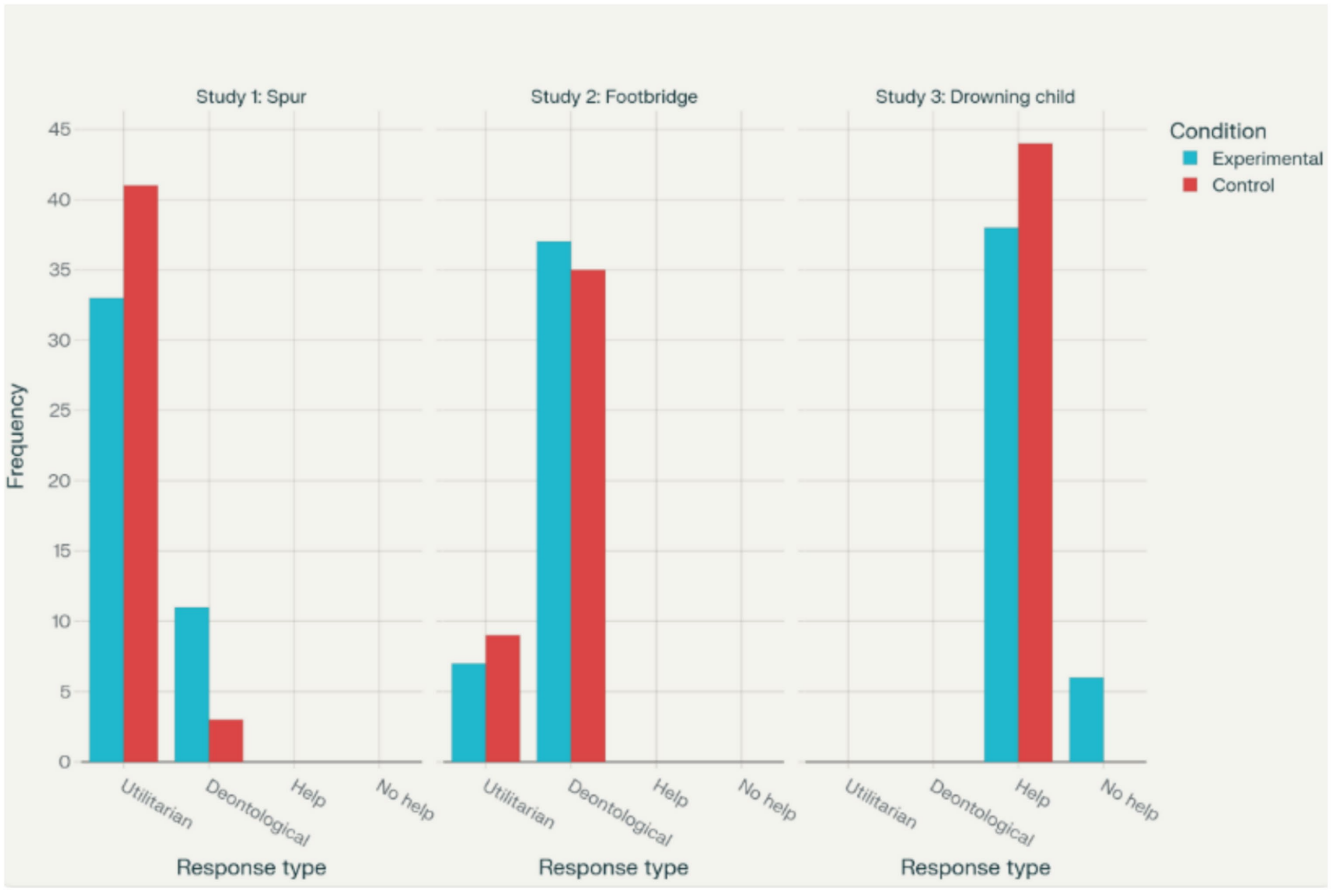
Frequencies of responses to dilemmas.

Results of Fisher’s studies are presented in Figure 1. Both regression models are presented in Table 1.

**Table 1:** Logistic regression models.

Study	Outcome (reference)	Predictor	Estimate (log-odds)	SE	Z	*p*	Odds ratio (95% CI)	Pseudo-*R^2^* (McFadden)
Study 1	Non-utilitarian *vs.* utilitarian	Intercept	−1.9952	2.384	−0.837	0.403	0.136 (0.00127–14.542)	0.0772
		Condition (control *vs.* experimental)	−1.4753	0.698	−2.114	0.034	0.229 (0.05826–0.898)	
		Age	0.0416	0.102	0.410	0.682	1.042 (0.85431–1.272)	
		Gender (female *vs.* male)	−0.1513	0.610	−0.248	0.804	0.86 (0.26007–2.841)	
Study 3	No *vs.* yes (save the child)	Intercept	−2.23288	4.810	−0.464	0.643	0.107 (8.62–1333.07)	0.205
		Condition (control *vs.* experimental)	−18.75177	2664.470	−0.007	0.994	7.18 (0–inf)	
		Gender (male *vs.* female)	0.42683	0.882	0.484	0.628	1.532 (0.272–8.62)	
		Age	0.00880	0.214	0.041	0.967	1.009 (0.663–1.53)	

The discrepancy between the significant Fisher’s exact test and the non-significant logistic regression in Study 3 stems from small cell counts and complete separation in the control group. Fisher’s exact test, a non-parametric method based on the exact sampling distribution, remains valid even when one cell is very small or zero. Logistic regression, however, performs poorly under these conditions: the zero cell for the “no-help” outcome caused complete separation, yielding an extreme coefficient (Estimate = −18.75, SE = 2664.47, *p* = 0.994) and an unstable *z*-statistic. Following the analytic plan and recognizing this limitation, interpretation relied on Fisher’s test, treating the manipulation as significantly associated with willingness to save the child, while noting that regression estimates were unreliable due to sparse data.

## Discussion

The present findings contribute to ongoing debates about the psychological impact of belief in free will and its role in moral cognition. Contrary to expectations based on previous research on moral dilemmas, the manipulation of belief in free will produced significant differences in the impersonal Spur trolley dilemma but not in the more affectively charged Footbridge variant. Typically, the opposite pattern is observed in trolley research: experimental interventions tend to shift responses in the Footbridge condition, where moral decisions are personal and emotionally salient, while the Spur scenario tends to produce stable utilitarian judgments with little variability across conditions (Cao et al., 2017; Gosling and Trémolière, 2021; Greene, 2014). That the current manipulation primarily affected the Spur case, with a parallel reduction in helping in the Singer dilemma but no detectable change in Footbridge, is consistent with the idea that disbelief in free will differentially affects these scenarios. However, because each dilemma was administered in a separate study, these cross-dilemma contrasts should be treated as provisional and in need of confirmation in a design that tests the interaction directly.

Conceptually, the present design captures binary behavioral choices—whether to intervene or not in the trolley scenarios, and whether to help or not in the Singer dilemma—rather than direct measures of underlying moral principles or processes. In the case of Spur, non-intervention can be read as deontological (non-utilitarian) in the standard trolley framework, but it is equally compatible with omission bias or reluctance to initiate harm, which aligns closely with the notion of moral inertia advanced here. Likewise, in the Singer scenario the key outcome is willingness to engage in prosocial helping rather than a clean utilitarian/non-utilitarian contrast, so any references to utilitarian or non-utilitarian perspectives should be understood as interpretive labels applied to observed choice patterns rather than as independently measured constructs.

In previous literature, variations in the Footbridge responses have been linked to both dispositional and situational factors. For instance, studies have found that intoxication (Duke and Bègue, 2015), cognitive load (Tinghög et al., 2016), and even using a foreign language (Andrade, 2022) can increase utilitarian responses—suggesting that emotional distance or reduced empathic engagement makes people more willing to endorse direct harm for a greater good. Personality constructs such as psychopathy or Machiavellianism also predict greater willingness to endorse the utilitarian option in the Footbridge dilemma, whereas such differences rarely appear in the more abstract Spur case (Nasello et al., 2023). Likewise, neuropsychological studies indicate that lesions in ventromedial prefrontal areas—regions associated with emotional regulation—disinhibit utilitarian responses mainly in the Footbridge scenario, leaving judgments in the Spur dilemma largely unaffected (Rowley et al., 2018). The consistency of these patterns across domains has contributed to the view that only personal, emotionally charged dilemmas such as Footbridge are sensitive to manipulations of cognitive or affective input, while the impersonal dilemmas are cognitively straightforward and yield uniform utilitarian reasoning.

From that perspective, the current results are anomalous: belief suppression influenced the impersonal, not the personal, version. Yet examining the cognitive meaning of belief in free will offers a possible explanation. Unlike manipulations that alter emotional engagement or cognitive control, weakening belief in free will modifies participants’ perceived epistemic and motivational stance toward agency itself. When people read deterministic arguments suggesting that every action unfolds according to prior neural causes, they may come to view human agency—including their own decision-making—as illusory or inconsequential. This altered self-perception does not necessarily encourage harm or cold rational calculation; instead, it can foster passivity and detachment, promoting a sense of reduced ownership over outcomes. The key feature in the Spur scenario is precisely the decision to intervene—pulling the lever requires an active step to change the course of events. A diminished sense of agency might render participants less inclined to intervene at all, leading them to accept the default trajectory of the trolley as something “already determined.”

In contrast, the Footbridge dilemma inherently centers on intentional harm—pushing a person into harm’s way—and thus vividly triggers normative prohibitions independent of one’s introspective sense of agency. Even if an individual’s belief in free will is weakened, the moral aversion to performing a direct physical transgression may persist robustly. Personal harm activates strong emotional circuits associated with empathic concern and aversive arousal (Greene et al., 2004). Such intuitive resistance requires high emotional override to overcome, and subtle changes in cognitive worldview may be insufficient to counter those entrenched affective responses. Consequently, disbelief in free will may have little leverage in the Footbridge condition, as non-utilitarian inhibition dominates the decision regardless of prior belief shifts.

From this interpretive perspective, the effect of the manipulation on the Spur dilemma suggests a distinct psychological mechanism—what could be called “moral inertia” (Maxwell, 1992; Sartorio, 2008). If determinism primes indeed make participants feel less like causal agents, the result may be a reluctance to disrupt the physical or moral status quo. The deterministic worldview implies that events are already unfolding according to inexorable laws; taking action might appear futile. Pulling the lever might thus feel like opposing inevitability rather than performing a rational moral intervention. As a result, those primed with determinism may simply refrain from acting, not because they adopt a non-utilitarian principle against intervention, but because they experience diminished volitional authorship over events. This aligns with broader findings from free-will disbelief studies showing reduced initiative, lower effort, and diminished prosocial behavior following deterministic primes (Baumeister et al., 2009; Caspar et al., 2017).

The “Consequences, norms, and generalized inaction” (CNI) model developed by Gawronski et al. (2017) offers a useful formal framework for unpacking the processes that may underlie the patterns observed in the present studies. Rather than treating “utilitarian” versus “deontological” (or non-utilitarian) choices as unitary constructs, the CNI approach decomposes responses to sacrificial dilemmas into three latent parameters: sensitivity to consequences for overall welfare (C), sensitivity to moral norms (N), and a general preference for inaction versus action irrespective of consequences and norms (I) (Gawronski and Ng, 2025). This decomposition addresses a central ambiguity of traditional trolley paradigms, namely that endorsement of an action option simultaneously implies maximizing outcomes and violating a norm, whereas choosing inaction implies the opposite, making it difficult to infer whether a given response reflects consequential concern, norm adherence, or an independent bias toward inaction.

Viewed through this lens, the current findings speak more directly to generalized inaction than to pure shifts in utilitarian or deontological tendencies. The reduced willingness to pull the lever in Spur and the small increase in refusal to save the child in the Singer scenario can be read as heightened I, that is, a stronger default toward omission, rather than as unequivocal evidence of increased N (a deontological commitment against intervention) or reduced C (indifference to outcomes). In other words, refusing the “utilitarian” option in these cases does not, by itself, demonstrate a deontological non-utilitarian judgment; consistent with the CNI critique, single binary choices in standard dilemmas cannot uniquely identify whether people are primarily tracking consequences, norms, or an action–omission preference. For this reason, the interpretation of “moral inertia” in this context is intentionally framed at the behavioral level (intervention vs. omission), with CNI-style constructs invoked as theoretically plausible, but not directly estimated, process-level accounts.

At the same time, the correspondence between the notion of moral inertia and the I parameter in the CNI model suggests concrete directions for future work. A natural next step would be to embed the free-will manipulation within a full CNI design that orthogonally varies consequences and norms, allowing estimation of separate C, N, and I parameters before and after determinism primes. Such a design would make it possible to test whether disbelief in free will selectively increases generalized inaction (I), leaves norm sensitivity (N) largely unchanged in high-transgression scenarios like Footbridge, and perhaps modestly alters sensitivity to consequences (C) in impersonal settings—thereby answering more precisely whether refusing a utilitarian option reflects deontological commitment, amplified inaction, or some combination of both.

This also helps clarify the partial parallel observed in Study 3, the Singer-style “Drowning child” dilemma. While helping a drowning child is normally a near-universal moral intuition, the small number of participants who refused to intervene after exposure to deterministic arguments might reflect the same mechanism of passivity observed in the Spur condition. The required act of saving the child demands spontaneous personal initiative rather than compliance with a prohibitive rule. In the absence of a strong internal sense of agency, participants may default to inaction, rationalizing that what happens is predetermined or outside their control. This “diffusion of moral agency” is not rooted in utilitarian calculation but in existential resignation—a belief that intervention cannot meaningfully alter outcomes. Philosophically, this resonates with the deterministic fatalism discussed by Nietzsche’s notion of *amor fati*—the acceptance of events as necessary (Nietzsche, 2001). Psychologically, it maps onto learned helplessness processes, in which perceived lack of control leads to behavioral withdrawal even when control is objectively possible (Maier and Seligman, 1976).

The asymmetry between the Spur and Footbridge effects could therefore be understood in terms of differential dependencies on perceived agency. The Spur task foregrounds an active decision to influence outcomes, whereas the Footbridge task foregrounds moral transgression. Suppressing free-will belief selectively attenuates moral motivation that depends on self-as-causal-agent awareness, while leaving intact affective prohibitions against directly harming others. This interpretation fits theories of moral cognition that distinguish between agentic and evaluative components. Cushman (2008) proposed that moral judgment involves both a “causal responsibility” process—deciding whether one sees oneself as the source of change—and an “outcome evaluation” process—judging whether the effects are acceptable. Determinism-related belief manipulations may primarily act on the first process, reducing causal responsibility attribution and thereby dampening the motivation to intervene.

A helpful way to interpret the asymmetry between reduced proactivity and unchanged willingness to cause harm is to distinguish initiative costs from transgression costs (Cushman et al., 2006). Determinism primes may selectively undermine the motivational resources needed to initiate action, which fits work linking disbelief in free will to reduced effort, self-control, and helping behavior. In contrast, directly harming another person engages strong prohibitive norms and affective alarms that are relatively insensitive to subtle shifts in abstract agency beliefs, so the “cost” of overcoming fear, guilt, and anticipated blame remains high even when people momentarily doubt their own freedom.

From this angle, one could say that intervention dilemmas like Spur and Singer primarily require rational endorsement of a low-cost, prosocial initiative, whereas Footbridge additionally requires overriding intense anticipatory emotions about being the author of harm. In contexts where the rational calculus and the emotional prohibitions both point toward inaction—as when doing nothing avoids personal involvement and direct transgression—disbelief in free will may simply tip people further toward omission and moral inertia. By contrast, when the only way to prevent severe harm is to commit a salient physical transgression, entrenched emotional barriers can dominate the decision, limiting the influence of short-term changes in agency beliefs on willingness to cause harm.

Prior work suggests, however, that disbelief in free will does not only promote passivity: in some settings it has been associated with increased aggression, especially when self-control is taxed or antisocial impulses are already activated (Carey and Paulhus, 2013). This implies that disbelief in free will might increase harmful behavior under conditions that (a) minimize fear of blame or detection, (b) frame aggression as low-effort or normatively permitted (e.g., following orders, acting within a group), or (c) reduce empathic concern, as in highly impersonal, dehumanizing, or punitive contexts. The present findings therefore fit a broader picture in which weakened free-will belief most reliably dampens prosocial initiative, but in combination with disinhibiting cues or hostile motivations it could, in principle, also remove internal brakes on causing harm—a possibility that future work should examine using paradigms that orthogonally manipulate agency beliefs, emotional prohibitions, and situational incentives for aggression.

An additional implication arises for moral psychology more broadly. The finding that belief in free will can reduce utilitarian responding in an impersonal context suggests that moral cognition is not purely a matter of reasoning style but also of existential stance. Traditional dual-process theories juxtapose utilitarian reasoning with non-utilitarian emotional intuition (Białek and Neys, 2017), but the current pattern implies a third influence—beliefs about agency mediating engagement itself. Participants with diminished free-will belief might not engage either system fully: they neither deliberate outcome trade-offs nor feel the emotional urgency of moral choice. Instead, they disengage from the process entirely, leading to indecision or omission.

Beyond cognitive interpretation, social and contextual factors might also amplify these effects. The use of Venezuelan student samples is a strength because most experimental work on free will and moral judgment has relied on Western, educated, industrialized, rich, and democratic (WEIRD) samples, and cross-cultural work suggests that beliefs about free will and control vary systematically across societies (Sarkissian et al., 2010). In recent years, prolonged periods of turmoil and hardship in Venezuela and other Latin American countries, including recurrent crises and shifting institutional landscapes, may have fostered chronic perceptions of external constraint, unpredictability, and learned helplessness at the societal level. In such settings, individuals can become accustomed to viewing key outcomes as shaped by distant, powerful forces, which may reinforce a baseline tendency toward omission and resignation when confronted with high-stakes decisions. This socio-historical backdrop may thus interact with experimentally induced disbelief in free will, jointly nudging people away from proactive intervention and toward moral inertia rather than toward more actively deontological or utilitarian engagement.

At the same time, regional political and moral discourse often oscillates between collectivist appeals to sacrifice for a “greater good” and strong denunciations of direct harm, corruption, or betrayal, creating a complex normative environment for moral reasoning. On one hand, rhetoric about necessary sacrifices for social change can evoke utilitarian framings; on the other hand, everyday experiences of institutional fragility and perceived impunity may undermine confidence that individual actions translate into just outcomes, weakening the motivational force of both deontological duties and utilitarian calculations. Within this milieu, inaction can become a psychologically coherent strategy, where refraining from intervention reflects not only omission bias but also a historically grounded skepticism about one’s capacity to effect positive change. Future work should therefore incorporate explicit measures of free-will beliefs, locus of control, structural constraint, and political efficacy to test whether these contextual factors in Venezuelan and other Latin American samples moderate the balance between deontological, utilitarian, and inertial tendencies.

It is important to point out that the present data do not directly measure cultural dimensions such as fatalism, locus of control, religiosity, or perceived structural constraint, so any link between those factors and the observed pattern must remain speculative. Rather than treating “Venezuelan context” as an explanation for the findings, it is therefore more appropriate to regard it as a motivation for future research that explicitly measures these constructs and tests whether they moderate the effects of determinism primes on moral inertia and intervention.

Neuroscientific perspectives also lend plausibility to this interpretation. Imaging studies show that the sense of agency involves a network including the supplementary motor area and the posterior parietal cortex, regions responsible for linking intentions with action outcomes (Haggard, 2017). When belief in agency is undermined, the neural prediction of one’s ability to change events may weaken, producing a subjective disconnection between intention and effect. Translating this to moral choice, participants experiencing reduced agency might perceive their decision as irrelevant to the causal flow of events—whether in pulling a lever or saving a drowning child. Their responses thus embody not moral indifference but perceived futility.

Taken together, the present findings indicate that disbelief in free will primarily reduces proactive moral agency rather than altering moral principles per se. The determinism prime did not make individuals more permissive of harm in the Footbridge scenario but made them less likely to intervene in situations requiring volitional initiation of action in the Spur and Drowning Child dilemmas. This sheds new light on how existential beliefs shape moral cognition: not merely by adjusting moral calculus between utilitarian and non-utilitarian options, but by modulating whether people experience themselves as participants or bystanders in the moral world. The paradoxical outcome—that deterministic thinking decreases intervention even when it would prevent harm—emphasizes that freedom of will, whether metaphysically real or not, functions psychologically as the foundation of moral responsibility and moral action.

## Limitations

The present research has some limitations. The sample sizes, though determined by power analysis, were modest and may limit generalizability. The trolley and Singer dilemmas, while widely used in moral psychology, are hypothetical and may not fully capture the complexity of real moral decisions. In addition, participants were recruited through convenience sampling rather than probabilistic methods, which could constrain representativeness. Future studies could address these issues by using larger, more diverse samples and by incorporating more ecologically valid scenarios, perhaps involving immersive or real-world decision-making contexts to better assess the effects of free-will belief manipulation. A further design limitation is that each dilemma was administered in a separate study and sample, so dilemma type is confounded with study and cohort. As a result, the present data cannot formally test an interaction between experimental condition and dilemma type, and cross-dilemma comparisons should be interpreted as suggestive patterns rather than as results of a single factorial design.

A further limitation concerns the absence of a manipulation check for the determinism prime. The studies did not include post-manipulation measures of free-will belief, determinism, or perceived agency/control, so the data cannot directly confirm that the Crick passage reduced belief in free will in this specific language and sample. As a result, the findings are best interpreted as effects of exposure to a determinism-themed text, with the free-will-belief mechanism remaining tentative rather than conclusively established. Future research should incorporate validated scales of free-will beliefs, locus of control, and perceived agency immediately after the prime in order to test whether changes in these constructs statistically mediate the observed pattern of reduced intervention versus omission.

A related limitation is that the dependent variables are dichotomous choices without process measures, so the studies cannot decisively distinguish between non-utilitarian rule adherence, omission bias, and more general reluctance to initiate harmful or effortful action. For this reason, the primary interpretation is framed in behavioral terms (intervention vs. omission, helping vs. not helping), with non-utilitarian and utilitarian language offered as plausible, but not uniquely supported, conceptual readings of these choices.

## Conclusion

The present findings point toward a specific way in which free-will beliefs matter for moral psychology: they seem to support engagement in moral action rather than simply shifting people between utilitarian and non-utilitarian judgments. In both the Spur and Singer-style dilemmas, the determinism manipulation reduced intervention in situations where action is relatively uncontroversial and clearly beneficial. By contrast, it did not increase willingness to endorse direct personal harm in the Footbridge case, where strong non-utilitarian prohibitions remain intact. This pattern suggests that weakened belief in free will does not create “cold” consequentialists, but instead fosters a tendency toward omission and moral inertia.

For future research, this implies that experimental work on free-will belief should move beyond asking whether people become more or less utilitarian, and instead systematically examine when people disengage from moral decision-making altogether. Designs that distinguish clearly between doing harm, allowing harm, and failing to help would be especially informative, as would measures of perceived control, self-efficacy, and counterfactual impact (“Would my choice really change anything?”). Longitudinal or field studies could test whether chronically low belief in free will predicts real-world bystander behavior, whistle-blowing, or civic participation—domains where the crucial question is whether one steps in or stands aside.

The results also suggest a fruitful bridge to literatures on motivation and learned helplessness. If disbelief in free will undermines the felt connection between intention and outcome, it may generalize beyond moral dilemmas to domains such as health behavior, political engagement, or organizational ethics. Future studies might therefore embed agency-belief manipulations in more ecologically rich tasks—team decisions, resource allocation games, virtual-reality emergencies—to see whether the same pattern of reduced initiative appears. Finally, the use of a non-WEIRD sample underscores the need for cross-cultural work on how baseline fatalism, structural constraint, and institutional trust moderate these effects. In that broader agenda, the current studies serve less as a definitive verdict on free-will belief than as a demonstration that even brief challenges to agency can measurably dampen people’s willingness to act when moral action is required.

## Data Availability

The raw data supporting the conclusions of this article will be made available by the authors, without undue reservation.
