# Supplementary material for: Free will and trolley dilemmas: evidence for moral inertia in a Venezuelan sample
Source: Front Psychol. 2026 Jan 30;17:1748028. doi: 10.3389/fpsyg.2026.1748028 (PMC12901468; doi:10.3389/fpsyg.2026.1748028)
Supplement: Supplementary file 1 [file Data_Sheet_1.pdf]

SUPPLEMENTARY MATERIALS

Spur 1 Dilemma:

Un tranvía fuera de control avanza por la vía principal y, si sigue su camino, atropellará y matará a cinco personas que se encuentran más adelante en la vía. Usted se encuentra junto a una palanca que puede desviar el tranvía hacia una vía secundaria. En esa vía secundaria hay una sola persona. Si usted acciona la palanca, el tranvía cambiará de vía y matará a esa persona, pero las cinco personas de la vía principal se salvarán. ¿Accionaría Ud. la palanca?

Footbridge Dilemma:

Imagine que un tranvía fuera de control avanza por la vía y, si continúa su curso, atropellará y matará a cinco personas que están más adelante. Usted se encuentra sobre un puente que cruza la vía, junto a un hombre muy corpulento. No hay palancas ni desvíos; la única manera de detener el tranvía y salvar a las cinco personas es empujar a este hombre desde el puente hacia la vía, de modo que su cuerpo detenga el vehículo, causando su muerte. ¿Debería usted empujar a este hombre para salvar a las cinco personas?

Singer’s dilemma:

Imagine que usted camina junto a un estanque poco profundo cuando ve a un niño que se está ahogando a pocos metros de la orilla. No hay nadie más cerca que pueda ayudar y, si usted no actúa de inmediato, el niño morirá. Usted lleva puestos unos zapatos muy caros, que se arruinarán si entra al agua para rescatarlo. Si se lanza al estanque, podrá salvar al niño, pero perderá sus zapatos. ¿Se metería usted al estanque para salvar al niño, aun sabiendo que estropeará sus zapatos costosos?

Crick’s text from *The Astonishing Hypothesis:*

El libre albedrío es, en muchos sentidos, un tema algo pasado de moda.

La mayoría de las personas lo dan por sentado, ya que sienten que, por lo general, son libres de actuar como desean. Aunque abogados y teólogos tal vez deban enfrentarse a esta cuestión, los filósofos, en su mayoría, han dejado de mostrar mucho interés en el asunto. Y casi nunca es mencionado por psicólogos o neurocientíficos. Algunos físicos y otros científicos que se preocupan por la indeterminación cuántica a veces se preguntan si el principio de incertidumbre no estará en el fondo del libre albedrío.

Yo mismo apenas había prestado atención al tema hasta 1986, cuando recibí una carta de un viejo amigo, Luis Rinaldini, un bioquímico argentino a quien conocí en Cambridge a fines de los años cuarenta. Luis y su esposa vivían entonces en Mendoza, una ciudad argentina a los pies de los Andes. Me escribió para decirme que viajaría a Estados Unidos y quería conversar conmigo sobre algunas de sus ideas. Cuando nos encontramos en Nueva York, me contó que él y un grupo de amigos habían formado un círculo de debate en Mendoza, y que últimamente se había interesado mucho por el tema del libre albedrío. Más tarde me escribió de nuevo, desarrollando sus reflexiones con más detalle.

Hasta ese momento, no era consciente de tener una teoría propia sobre el libre albedrío; sin embargo, al leer lo que él me escribía, noté que mis ideas diferían un poco de las suyas. Entonces redacté, muy brevemente, lo que descubrí que pensaba al respecto y se lo envié. El texto no ocupaba más de treinta líneas. Se lo mostré a la filósofa Patricia Churchland, en parte para asegurarme de no estar diciendo tonterías. Ella me ayudó a aclarar la redacción y añadió un punto adicional, comentando que mis ideas le parecían plausibles. Lo que sigue es una versión un poco más desarrollada de lo que le escribí a Luis.

Partí de la idea de que uno puede elaborar planes para acciones futuras, sin necesidad de llevarlos a cabo. También supuse que uno puede ser consciente de esos planes, es decir, que puede recordarlos de inmediato.

Mi segunda suposición era que no somos conscientes de los “cálculos” que realiza esa parte del cerebro, sino solo de las “decisiones” que toma —es decir, de sus planes. Esos cálculos, desde luego, dependen de la estructura de esa región cerebral (resultado en parte de factores epigenéticos y en parte de la experiencia previa) y de las señales que recibe de otras áreas del cerebro.

Mi tercera suposición era que la decisión de actuar según un plan u otro está sujeta a las mismas limitaciones. En otras palabras, podemos recordar de inmediato lo que se ha decidido, pero no los cálculos que llevaron a esa decisión, aunque seamos conscientes del plan de movimiento.

Un "mecanismo" así (esa fue la palabra que usé en mi carta) tendría la impresión de poseer libre albedrío, siempre que pueda atribuirse intencionalidad, es decir, que tenga una imagen de sí mismo.

La causa real de la decisión puede ser simple (como señaló Pat), o bien determinista pero caótica: una pequeña variación podría generar una gran diferencia en el resultado final. Eso daría la impresión de que la voluntad es "libre", ya que el resultado sería, en la práctica, impredecible. Además, los procesos conscientes pueden influir en el mecanismo de decisión (otro aporte de Pat).

Un sistema así podría intentar explicarse a sí mismo por qué tomó cierta decisión (mediante la introspección). A veces llegaría a la conclusión correcta; otras veces no sabría o, más probablemente, inventaría una explicación, ya que carece de conocimiento consciente de la "razón" de su elección. Esto implica que debe existir un mecanismo de confabulación: dado cierto conjunto de evidencias —que podrían o no ser engañosas—, una parte del cerebro tiende a saltar a la conclusión más simple. Como ya hemos visto, eso puede suceder con demasiada facilidad.

Así concluía mi "Teoría del Libre Albedrío". Obviamente depende de entender qué es la conciencia (tema central de este libro), cómo el cerebro planifica y ejecuta acciones, cómo confabulamos, etc. Dudo que haya algo realmente nuevo en todo esto, aunque algunos detalles quizá no se hayan presentado de este modo antes.

Y así dejé el asunto descansar. Me reuní con Luis en Nueva York, y más tarde él visitó La Jolla, en California. También pudo discutir el tema con Paul Churchland, esposo de Patricia. No tenía intención de seguir pensando sobre el tema, pero una vez despertado mi interés, me descubrí reflexionando sobre ello de vez en cuando.

Me preguntaba: ¿dónde podría ubicarse el libre albedrío en el cerebro? Es evidente que implica la interacción de varias áreas, pero no era descabellado pensar que una parte de la corteza podría estar especialmente implicada. Sería lógico esperar que reciba información de los niveles superiores de los sistemas sensoriales y que esté conectada con las áreas de planificación del sistema motor.

En ese momento, me topé con un informe de Antonio Damasio y sus colegas sobre una mujer con daño cerebral localizado. Después de la lesión, parecía muy poco reactiva: permanecía tranquila en la cama, con expresión alerta, seguía con la mirada a las personas pero no hablaba espontáneamente. No respondía verbalmente a las preguntas, aunque parecía comprenderlas, ya que asentía con la cabeza. Podía repetir palabras y frases, pero muy lentamente. En resumen, sus reacciones eran limitadas y algo estereotipadas.

Un mes después, se había recuperado en gran medida. Contó que no se había sentido angustiada, simplemente porque había sido incapaz de comunicarse: podía seguir las conversaciones, pero no hablaba porque "no tenía nada que decir". Su mente había estado "vacía". Inmediatamente pensé:

“ha perdido su voluntad”. ¿Dónde estaba la lesión? Resultó estar en o cerca de una región llamada “surco cingulado anterior”, junto al área 24 de Brodmann. Es una zona interna del cerebro —visible si se corta en dos mitades— hacia el frente (de ahí lo de “anterior”) y cerca de la parte superior. Me alegró descubrir que esa región recibe numerosas señales de las áreas sensoriales superiores y está relacionada con los niveles más altos del sistema motor.

En el Instituto Salk, el grupo de Terry Sejnowski suele reunirse para tomar té por las tardes. Esas reuniones son perfectas para comentar los últimos resultados experimentales, compartir ideas o simplemente charlar sobre ciencia y actualidad. Un día, durante el té, anuncié a Pat Churchland y a Terry que ¡había encontrado el asiento de la voluntad! Estaba en o cerca del surco cingulado anterior.

Cuando hablé del asunto con Antonio Damasio, descubrí que él también había llegado a la misma conclusión. Me explicó algunas de las conexiones anatómicas de esa zona del cerebro: tiene vínculos fuertes con la región correspondiente del otro hemisferio —por lo general, solo tenemos una única “voluntad” en un momento dado, aunque, como vimos en el capítulo 12, los cerebros divididos pueden tener dos—. Además, esa zona se conecta de manera significativa con el cuerpo estriado (una parte esencial del sistema motor) de ambos lados del cerebro, algo esperable si realmente existe una sola voluntad. Era una hipótesis muy prometedora.

Tiempo después, leí un artículo de Michael Posner que mencionaba un curioso trastorno causado por cierto tipo de daño cerebral: el “síndrome de la mano ajena”. En este caso, la mano izquierda del paciente, por ejemplo, puede realizar movimientos simples y automáticos que la persona niega haber iniciado. A veces la mano agarra un objeto colocado cerca, y el paciente es incapaz de hacer que lo suelte, debiendo usar la otra mano para separarla. Un paciente descubrió que no podía lograr que su “mano ajena” lo soltara mediante su voluntad, pero sí lograba que obedeciera si le decía en voz alta: “¡suéltalo!”.

¿Y dónde estaba la lesión? De nuevo, en o cerca del surco cingulado anterior (en el lado derecho, tratándose de una mano izquierda “ajena”), pero también en parte del cuerpo calloso, de modo que la región izquierda no podía enviar instrucciones a la mano que la parte derecha dañada ya no podía controlar. Además, como se mencionó en el capítulo 8, el cingulado anterior se activa durante ciertos procesos de selección, algo evidenciado por el aumento del flujo sanguíneo en esa zona.

Quizás este aspecto sea realmente novedoso: el libre albedrío estaría localizado en o cerca del surco cingulado anterior. En la práctica, las cosas seguramente son más complejas. Otras áreas frontales del cerebro podrían participar también. Harían falta más experimentos con animales, el estudio detallado de más casos de “mano ajena” y, sobre todo, una comprensión neurobiológica más profunda de la conciencia visual y, a partir de ella, de otras formas de conciencia. Por eso incluyo esta sugerencia al final de este libro.

Text for the control group:

El cerebro está formado por alrededor de ochenta y seis mil millones de neuronas, acompañadas por casi la misma cantidad de células gliales, que le brindan soporte, nutrición y estabilidad. Cada neurona puede establecer miles de conexiones, llamadas sinapsis, mediante las cuales se comunican entre sí a través de impulsos eléctricos y señales químicas. Estas conexiones constituyen redes inmensas y dinámicas, que cambian con la experiencia, el aprendizaje y el paso del tiempo. Así, el cerebro no es una estructura fija, sino un sistema vivo y adaptable, moldeado por la interacción entre la biología heredada y el entorno en que cada persona se desarrolla.

En términos anatómicos, la parte más externa y evolutivamente reciente del cerebro es la corteza cerebral, una capa de unos pocos milímetros de espesor que cubre los hemisferios cerebrales como un manto plegado. Su superficie ondulada permite aumentar su área total sin ocupar demasiado volumen, lo que ha otorgado a los seres humanos una capacidad extraordinaria para procesar información. Cada pliegue visible en la corteza —los surcos y circunvoluciones— marca territorios funcionales que colaboran de manera jerárquica y distribuida.

Tradicionalmente, se divide la corteza en cuatro lóbulos principales. El lóbulo frontal, situado en la parte anterior del cerebro, es el que más se asocia con las funciones ejecutivas: planificación, atención, organización y control de la conducta. Es la región que nos permite formular metas, anticipar consecuencias y ajustar nuestras acciones a contextos cambiantes. Muy cerca de su zona posterior se encuentra la corteza motora primaria, que envía las órdenes hacia los músculos del cuerpo para ejecutar los movimientos voluntarios.

Detrás del surco central empieza el lóbulo parietal, encargado de integrar la información procedente de los sentidos, especialmente el tacto, la propiocepción y la percepción espacial. Es aquí donde el cerebro construye un mapa continuo del cuerpo y del entorno, lo que nos permite orientarnos, manipular objetos y coordinar nuestros movimientos con precisión.

En la parte inferior, el lóbulo temporal alberga áreas cruciales para la audición y la memoria. En su interior se encuentra el hipocampo, una estructura con forma de caballito de mar que desempeña un papel esencial en la formación de nuevos recuerdos y en la consolidación de la memoria a largo plazo. El lóbulo temporal también interviene en la comprensión del lenguaje y en la percepción de los significados simbólicos, lo que lo convierte en un puente entre la experiencia sensorial y el pensamiento abstracto.

En la parte posterior del cerebro se ubica el lóbulo occipital, donde se localiza la corteza visual primaria. Allí llegan las señales provenientes de los ojos, que el cerebro traduce en formas, colores y movimiento. Sin embargo, la visión no se limita a esta zona: las imágenes se proyectan hacia otras áreas corticales, donde se integran con la memoria, la atención y la emoción, permitiendo reconocer rostros, percibir profundidad o imaginar escenas inexistentes.

Más allá de la corteza, el cerebro contiene regiones subcorticales fundamentales. El tálamo, situado en el centro, actúa como una estación de retransmisión: casi toda la información sensorial pasa por él antes de alcanzar la corteza. El hipotálamo, a su vez, regula muchas de las funciones automáticas del cuerpo: el hambre, la sed, la temperatura, el sueño y la respuesta al estrés. Aunque su tamaño es pequeño, su papel es crucial porque mantiene la homeostasis interna, comunicándose con la hipófisis para controlar la secreción hormonal.

Otro conjunto vital de estructuras subcorticales es el de los ganglios basales, circuitos profundos que participan en la coordinación del movimiento y en el aprendizaje de hábitos y secuencias motoras. Junto al cerebelo, que se encuentra en la parte posterior inferior del cráneo, son esenciales para el equilibrio, la precisión y la automatización de gestos y destrezas. El cerebelo también contribuye al procesamiento del tiempo y al ajuste fino de las acciones cognitivas y emocionales, algo que solo recientemente la ciencia ha comenzado a comprender.

El tronco encefálico, situado en la base del cerebro, conecta con la médula espinal y gobierna las funciones más básicas para la vida: la respiración, el ritmo cardíaco, la presión arterial y la vigilia. Allí se encuentran núcleos tan antiguos como los que generan la dopamina, la serotonina y la noradrenalina, neurotransmisores esenciales para la atención, la motivación y el equilibrio emocional. Sin esta región, la conciencia no podría sostenerse.

El cerebro no solo está dividido en regiones, sino también en hemisferios. Aunque popularmente se suele hablar de un "cerebro derecho" creativo y un "cerebro izquierdo" lógico, en realidad esta separación es más sutil. El hemisferio izquierdo suele destacar en el análisis secuencial, el lenguaje y la aritmética, mientras que el derecho participa más activamente en el reconocimiento de patrones, la música, las emociones y la orientación espacial. Lo relevante no es la diferencia entre ambos, sino la comunicación constante que mantienen a través del cuerpo calloso, un grueso haz de fibras que sincroniza la actividad entre las dos mitades.

Todo el cerebro funciona gracias a un equilibrio dinámico de excitación e inhibición. Cuando una neurona se activa, envía impulsos eléctricos que viajan por su axón hasta llegar a las sinapsis, donde se liberan neurotransmisores hacia la siguiente célula. Cada sinapsis puede fortalecer o debilitarse según la experiencia, un fenómeno conocido como plasticidad sináptica, que constituye la base biológica del aprendizaje y la memoria. De esta forma, cada nueva experiencia modifica sutilmente la arquitectura cerebral, reforzando ciertas conexiones y eliminando otras menos útiles.

Las células gliales, durante mucho tiempo consideradas simples auxiliares de las neuronas, hoy se reconocen como participantes activas en los procesos cognitivos. Los astrocitos regulan la composición química del entorno cerebral y modulan la transmisión sináptica; los oligodendrocitos forman las vainas de mielina que aíslan los axones y permiten que los impulsos viajen con mayor rapidez; y la microglía actúa como sistema inmunológico interno, vigilando y reparando daños. Sin ellas, el cerebro no podría mantener su extraordinaria eficiencia ni su capacidad de adaptación.

A nivel funcional, el cerebro puede entenderse como una red de sistemas interconectados que trabajan de manera simultánea. Los sistemas sensoriales recogen la información del entorno —luz, sonido, temperatura, movimiento— y la envían a centros especializados para su análisis. Los sistemas de atención seleccionan los estímulos relevantes y descartan los irrelevantes, mientras que los sistemas de memoria los codifican y almacenan. Todo esto ocurre en cuestión de milisegundos, en una danza constante de señales eléctricas que atraviesan miles de millones de sinapsis.

La cognición —ese conjunto amplio de procesos que incluye percibir, entender, recordar, imaginar y razonar— surge de la coordinación entre estas redes. El cerebro no procesa información como una máquina digital; su actividad es paralela, fluida y contextual. Por ejemplo, reconocer una palabra implica la cooperación de áreas visuales, fonológicas, semánticas y emocionales que trabajan a la vez. Las imágenes mentales, los recuerdos y las emociones impregnan cada acto cognitivo, de modo que pensar nunca es un proceso puramente lógico, sino profundamente integrado.

Las emociones desempeñan un papel central en este entramado. Estructuras como la amígdala y el sistema límbico evalúan la relevancia emocional de los estímulos y preparan al organismo para responder. Las emociones no son simples reacciones, sino mecanismos complejos de adaptación que influyen en la atención, la memoria y la toma de decisiones. La serotonina, la dopamina y otras sustancias químicas regulan el tono emocional general, ayudando a mantener el equilibrio entre entusiasmo, alerta y serenidad.

Resulta fascinante observar cómo el cerebro combina estabilidad y cambio. Sus conexiones básicas se establecen durante el desarrollo fetal y la infancia, pero la experiencia continúa esculpiendo sus redes durante toda la vida. La neuroplasticidad no desaparece con la madurez; se manifiesta cuando aprendemos un idioma, adquirimos una nueva habilidad o nos recuperamos de una lesión. Incluso en el envejecimiento, el cerebro mantiene una sorprendente capacidad de reorganización funcional.

En los últimos años, el avance de las técnicas de neuroimagen —como la resonancia magnética funcional o la tomografía por emisión de positrones— ha permitido observar el cerebro en acción. Estas herramientas han confirmado que los procesos mentales dependen de redes distribuidas más que de "centros" aislados: leer una frase, recordar una melodía o resolver un problema matemático implica la activación coordinada de múltiples regiones, que se comunican en patrones específicos de sincronía.

Aun así, siguen existiendo grandes enigmas. Sabemos mucho sobre las estructuras y los procesos, pero todavía ignoramos cómo de esos circuitos físicos emerge la experiencia consciente, o cómo la actividad neuronal se traduce en pensamientos y significados. La ciencia del cerebro avanza con cautela, consciente de que comprender el funcionamiento cognitivo requiere integrar la biología con la psicología, la lingüística y la filosofía.

Lo que sí parece claro es que la mente humana es el resultado de la cooperación entre millones de procesos microscópicos que ocurren cada segundo en nuestro interior. Cada percepción, recuerdo o idea es el reflejo de la actividad sincronizada de neuronas y glías, de neurotransmisores y redes cerebrales que se comunican sin cesar. Desde este punto de vista, pensar, sentir y actuar no son habilidades separadas, sino distintas manifestaciones de un mismo sistema en movimiento continuo.

El cerebro, en definitiva, es un universo biológico en miniatura: un espacio donde la materia genera significado, donde lo físico y lo mental se entrelazan de manera indisoluble. Su estudio no solo amplía nuestro conocimiento científico, sino que también nos invita a reflexionar sobre lo que somos. Comprender su estructura y sus mecanismos de funcionamiento no reduce la experiencia humana, sino que la enriquece, mostrándonos la sorprendente profundidad de los procesos que hacen posible la percepción, el pensamiento, la emoción y la conciencia de estar vivos.
